# Physical Activity Maintenance: A Critical Narrative Review and Directions for Future Research

**DOI:** 10.3389/fpsyg.2021.725671

**Published:** 2021-09-06

**Authors:** Ryan E. Rhodes, Wuyou Sui

**Affiliations:** Behavioural Medicine Laboratory, Physical and Health Education, School of Exercise Science, University of Victoria, Victoria, BC, Canada

**Keywords:** exercise, physical activity continuation, physical activity adoption, habit, identity, satisfaction, theories of physical activity

## Abstract

A clear rationale can be made for promoting long-term regular physical activity (PA), yet despite some attempts to operationalize “maintenance,” no robust definition has been agreed upon, beyond arbitrary time frames of regular PA. This has likely impaired the advancement of theory and practice. The purpose of this critical narrative review was to first overview the conception of maintenance and co-requisite theoretical constructs in theories used in PA research. Our subsequent aims were to engage in a critical analysis of this literature to propose a working definition of PA maintenance followed by recommendations for future research. Relevant behavioral theories were parsed for references to maintenance or maintenance-specific constructs and constructs most likely associated with maintenance were overviewed from a recent systematic review. Based on this information, we suggest PA maintenance be operationalized as a process marked by a shift in the mechanisms of action determining behavioral performance, that engender greater perceived behavioral enactment efficiency. We suggest that maintenance should not be considered an absolute state of behavioral performance (e.g., a stage), as some constructs that were critical to behavioral performance during initiation will still be critical during PA continuation. Based on this definition, we propose a method of falsifiability hypothesis testing of theoretical constructs that may determine the maintenance process. Finally, the review concludes with suggestions for future research using this operationalization of maintenance including measure development, tests of latency to reach the peak maintenance process, validating constructs critical to determining maintenance, exploration of the contextual and individual moderators of maintenance formation, and the development of an omnibus dynamic model of initiation, continuation, and maintenance in PA behavior change.

Regular physical activity, performed at a moderate-intensity or higher for 150 min or more per week, is associated multiple health benefits among adults (Rhodes et al., 2017). These benefits include, but are not limited to, reduced chances of all-cause mortality, heart disease, several cancers, obesity, type 2 diabetes, depression, and anxiety in the range of 30–40% (Warburton and Bredin, 2017). Cardiorespiratory and musculoskeletal fitness, academic achievement, and cognition, pro-social behaviors, metabolic health, and overall mental health benefits of daily moderate or higher intensity physical activity are also well-established for children and youth (American College of Sports Medicine, 2016; Poitras et al., 2016; Ahn et al., 2018; Biddle et al., 2019). Despite evidence that these health benefits have been widely recognized by most of the population for decades (e.g., Martin et al., 2000), many adults (Guthold et al., 2018) and youth (Guthold et al., 2019) do not meet these recommended physical activity guidelines. This high prevalence of inactivity has led to the development and application of many behavioral theories focused on physical activity and subsequent attempts to promote physical activity-related behavior change (Rhodes et al., 2019).

One of the interesting characteristics of the physical activity and health linkages noted above is the importance of performing the behavior *regularly*. Specifically, health promoters are interested in getting inactive people to *initiate* physical activity and then subsequently *maintain* the behavior across the lifespan (Laitakari et al., 1996). Not surprisingly, this aim has produced inquiry into both physical activity initiation and maintenance since very early on in the behavioral science literature, when researchers documented large (>50%) drop-out rates within the first 6-months among those beginning a physical activity program (Oldridge, 1984; Dishman et al., 1985; Sallis et al., 1986). The early interest in physical activity initiation vs. maintenance has persisted to the present, fueled by a continually growing evidence base that suggests there is a high drop-out of initiated physical activity patterns (Kahlert, 2015; McEwan et al., 2020). In fact, the phenomenon is so readily apparent, that it has general public awareness. For example, New Years exercise resolutions that fail soon after are a trope at this point in popular culture (Rhodes et al., 2020), and yearly gym memberships that go unused are featured as jokes in television sitcoms. Even commercial gyms, whose purpose is ostensibly to provide consumers with further opportunity to be more physically active, predicate their business model on the high drop-out rates among new exercise initiates (Smith, 2014).

It is clear that there are differences between physical activity initiation and maintenance, both epidemiologically and conceptually. However, despite several attempts to definitively operationalize “physical activity maintenance,” a robust definition of physical activity maintenance has not been agreed upon—a fact that likely impairs advancement of theory and practice (Kahlert, 2015). One way to conceive maintenance that differentiates it from initiation is the temporal component of behavioral performance. Specifically, maintenance involves behavior performed over time. Still, basing maintenance on a strictly temporal criterion (e.g., 6 months) has been duly criticized for being arbitrary and neglecting the individuality of the behavior change process (Bandura, 1997; Davidson, 2001). The passage of time alone is thus insufficient to qualify maintenance and a more precise conceptualization is needed.

The purpose of this paper is to provide a critical overview of the conceptions of maintenance in past theoretical research relevant to physical activity behavior. To achieve this purpose, we have four aims. Specifically, we (1) overview the conception of maintenance stated in many theories used in physical activity behavior research, as well as (2) highlight theoretical constructs that may discriminate initiation from maintenance. Based on this information, our subsequent aims are (3) to engage in a critical analysis of this literature in order to propose a working definition of physical activity maintenance and (4) recommend an agenda for future research to advance understanding on the differences between physical activity initiation and maintenance.

## Conceptions of Maintenance in Theories of Physical Activity

We adopted a critical narrative review methodology to overview current theories and conceptions of maintenance. According to Baethge and colleagues, narrative reviews “attempt to summarize the literature in a way which is not explicitly systematic” and may be better suited to addressing broader issues within a field, compared to systematic reviews (Baethge et al., 2019). As the aim of our review was to critique, as well as summarize, a critical narrative review was deemed appropriate for the purpose of this paper.

Eligible papers for our critical narrative review included, at least one of the following: (1) a conceptualization of maintenance from the major theoretical frames noted by Rhodes et al. (2019); (2) specific physical activity theories that have included a discussion of maintenance; (3) conceptual papers that have addressed or proposed a definition of physical activity maintenance; or (4) theoretical papers from health behavior psychology that specifically address maintenance pertinent to physical activity behavior.

The initial selection of behavior theories was based on the narrative synthesis. As such, we applied seven broad theoretical categories: 1. Social Cognitive Approaches; 2. Stage Models; 3. Humanistic Approaches; 4. Dual-Process Approaches; 5. Action Control Models; 6. Socioecological Frameworks; and 7. Other Schematics. Further, because our critical narrative review was focused specifically on maintenance, we engaged in searches for systematic reviews, commentaries, and recent literature in the first five pages of Google Scholar, PubMed, and Scopus databases with search terms (“physical activity” AND (“maintenance” OR “behavior change”) AND (“theory” OR “definition”); these papers and reviews were then parsed for behavior theories and conceptualizations of maintenance. If a theory or conceptualization was cited by a paper, the original paper was searched for and included in the review, if relevant. Given the focus of this paper was to offer a critical narrative review of the physical activity maintenance theory literature, rather than a systematic review, this approach was deemed sufficient to capture the most relevant theories and conceptualizations of physical activity maintenance (see Supplementary Figure 1).

In total, 20 theories and conceptual papers on physical activity maintenance were identified through this method. Reporting of these results was informed by the Scale for the Assessment of Narrative Review Articles (SANRA; Baethge et al., 2019 see Supplementary Table 1).

Table 1 details the results of our search of theories, conceptual papers, and relevant commentaries in physical activity research and the corresponding conception of maintenance. Historically, the most common theoretical approach to understanding physical activity has been using the social cognitive tradition (Rhodes et al., 2019). As referents of this tradition, we include discussion of maintenance from the perspective of theory of planned behavior (Ajzen, 1991), social cognitive theory (Bandura, 1986), and protection motivation theory (Rogers, 1983). None of these theories delineate initiation from maintenance; instead, the same operational determinants of behavior (e.g., outcome expectations, perceived control/self-efficacy) under the same structural conditions (e.g., mediated through intentions/goals) are expected to determine behavior in perpetuity. The exception to this approach in the social cognitive domain is the physical activity maintenance model (Nigg et al., 2008). While maintenance itself is not explicitly defined from initiation in this model, it positions maintenance as an active process requiring consistent self-regulation that involves goal setting, motivation and self-efficacy and highlights the potential environmental and individual triggers of relapse.

**Table 1:** Definition and operationalization of maintenance according to behavioral theories.

**Behavioral theory/conceptualization**	**Definition and/or reference to maintenance**
**Social cognitive approaches**	
Physical activity maintenance model (Nigg et al., 2008)	No explicit definition of maintenance. Long-term maintenance is described as an active process requiring active utilization of strategies and techniques for continued adherence to physical activity.
Theory of planned behavior (Ajzen, 1985, 1991)	No explicit definition of maintenance or delineation between the initiation of behavior and maintenance of behavior. Successful performance of a behavior - sustained or otherwise - is a direct function of the intention to perform said behavior.
Protection motivation theory (Rogers, 1975; Maddux and Rogers, 1983)	No explicit definition of maintenance or delineation between the initiation of behavior and maintenance of behavior. The initiation and maintenance of a behavior (i.e., an “avoidant response” to fear) is achieved through strong intention(s) to adopt/sustain the behavior.
Social cognitive theory (Bandura, 1986, 2004)	No explicit definition of maintenance or delineation between the initiation of behavior and maintenance of behavior. Reference to the temporal significance of some constructs (e.g., knowledge creates the “pre-condition” for behavior change).
**Stage models**	
Transtheoretical model (TTM; Marcus and Simkin, 1994; Prochaska and Velicer, 1997; Marshall and Biddle, 2001)	Maintenance is described as “the stage in which people are working to prevent relapse but they do not apply change processes as frequently as do people in action” (Prochaska and Velicer, 1997). This stage is expected to occur at approximately 6 months post- action phase, and last up to 5 years. Behavior change occurs (or is discontinued/relapsed) through a series of sequential stages. These stages are: (1) precontemplation; (2) contemplation; (3) preparation; (4) action; and (5) maintenance. Within the TTM, succession from one stage to the next requires targeting certain processes of change specific to that stage. No processes of change are described specific to sustaining maintenance of behavior.
The four phases of the behavior change process (Rothman et al., 2011)	Maintenance of behavior is characterized by the desire and sustained effort to continue a newly established pattern of behavior. Transition into the maintenance phase is marked by consistent performance of the desired behavior and complete confidence in one's ability to perform the behavior. Similarly, the shift from concerns with performing the behavior to evaluation of satisfaction with the new behavior marks the transition from this phase into habit.
**Humanistic approaches**	
Self-determination theory (SDT; Deci and Ryan, 2000; Ryan et al., 2008)	No explicit definition of maintenance or delineation between the initiation of behavior and maintenance of behavior. The constructs that aid individuals in acquiring the motivation to initiate a behavior (i.e., autonomy, competence, social relatedness) are thought to extend to the maintenance of the behavior by contributing to the process of internalization and integration. Specifically, increased internalization and integration results in a shift from the controlled motivations of behavior initiation (e.g., external regulation) to more autonomous (i.e., intrinsic) motivations expected from sustained behavior.
**Dual-process approaches**	
The physical activity adoption and maintenance model (PAAM; Strobach et al., 2020)	No explicit definition of maintenance. The model focuses less on an isolated modeling of adoption and maintenance of physical activity, in favor of physical activity behavior as a product of implicit and explicit processes. Behavior development over time is thought to increasingly rely on implicit processes (e.g., habit, affect) for behavior regulation, coupled with a reciprocal decrease in reliance on explicit processes (e.g., executive functions, trait self-regulations).
The affective-reflective theory of physical inactivity and exercise (Brand and Ekkekakis, 2018)	No explicit definition of maintenance or delineation between the initiation of behavior and maintenance of behavior.
Theory of energetic cost minimization (Cheval et al., 2018; Brand and Cheval, 2019)	No explicit definition of maintenance or delineation between the initiation of behavior and maintenance of behavior.
2 × 2 Matrix (Rothman et al., 2009)	The transition from behavioral initiation to behavioral maintenance is marked, in part, by a shift in decisional criteria from “what will happen” (e.g., outcome expectancies) to “what has happened” (e.g., satisfaction with outcomes).
Affect and health behavior framework (Williams and Evans, 2014)	No explicit definition of maintenance. Rather, both behavioral intention and maintenance are affected by automatic and reflective affective constructs. Successful repeated performances of a health behavior that elicit a positive affective response promote future behavioral performance through improving automatic affective associations and anticipated affective response(s) to the health behavior.
Maintain IT model (Caldwell et al., 2018)	Behavioral maintenance involves a diminished reliance upon the executive function resources involved in behavioral enactment as a result of the emergence of identities related to the behavior that facilitate swift and less effortful enactment (i.e., centered identity transformation).
**Action control models**	
Health action process approach (HAPA; Schwarzer, 2008; Schwarzer and Luszczynska, 2008)	No explicit definition of maintenance, but post-intentional constructs (i.e., action planning, coping planning, maintenance self-efficacy, recovery self-efficacy) are thought to be stronger amongst individuals who maintain behavior, compared to those who have only initiated a behavior.
Multi-Process action control approach (M-PAC; Rhodes, 2017)	No explicit definition of maintenance. Rather, maintenance is described as a result of the development of identity and habit (known as reflexive processes) and their subsequent determination of behavior over time.
The MoVo process model (Fuchs et al., 2012)	No explicit definition of maintenance or delineation between the initiation of behavior and maintenance of behavior. Rather, goal intention, along with volitional (i.e., post-intentional) constructs like action planning and barrier management are expected to be higher among individuals who can successfully maintain their behavior, compared to those who have only initiated physical activity.
**Socioecological frameworks**	
The Ecological Model of Physical Activity (Spence and Lee, 2003)	No explicit definition of maintenance or delineation between the initiation of behavior and maintenance of behavior. Rather, physical activity behavior in general is thought to be influenced by both psychological factors (e.g., attitudes, self-efficacy) and a cascading series of four ecosystems (i.e., microsystem, mesosystem, exosystem, and macrosystem), each of which encompass dimensions from increasingly distal social and physical environments to the individual.
**Other schematics**	
Timescale separated model (Spruijt-Metz et al., 2015)	No explicit definition of maintenance or delineation between the initiation of behavior and maintenance of behavior. Rather, long-term physical activity constructs (e.g., self-identity as an exercise, context-contingent habits) are thought to be influenced and shaped by the accrual of repeated short-term physical activity successes (e.g., meeting daily MVPA goals, daily/weekly exercise self-efficacy, momentary cues to action).
Lapse-recovery relationship (Kahlert, 2015)	Maintenance of physical activity is demarked by consistent “recoveries” from “lapses” of the “personal goal” of the individual, as well as a decline in the number of lapses over time. Specifically, the lapse-recovery approach views lapses and recoveries from achieving personal goals as indicators of physical activity maintenance. Demonstration of multiple lapses of personal goals without accompanying recovery may indicate that an individual is not yet maintaining physical activity, independent of time or prior behavior.
Successful maintenance of physical activity vs. maintenance of physical activity change (Marcus et al., 2000)	Successful physical activity maintenance is based on physical activity behavior, and distinct from successful maintenance of physical activity behavior *change*. Specifically, individuals who engage in physical activity at least 6 months post-intervention would have successfully maintained their physical activity *change*. By contrast, sedentary individuals who increase and perform regular physical activity for at least 6 months are considered to have successfully maintained their physical activity.

Stage models, such as the trans theoretical model of behavior change (Prochaska and Velicer, 1997), have also had a historical application in physical activity theory research (Marshall and Biddle, 2001; Rhodes and Nigg, 2011). In this theoretical approach, there is a formal demarcation of maintenance as a stage that occurs after behavior had been performed consistently for 6 months. The stage is also defined by a lessening of the application of behavioral processes of change (i.e., the behavioral tactics to enact a behavior) compared to initiation (known as the action stage), yet no specific processes or determinants are associated with the maintenance stage itself. The four phases of behavior change process created by Rothman et al. (2011), is a stage-based approach that also specifically outlines maintenance. In this approach, maintenance is described as the desire and sustained effort to continue a newly established pattern of behavior and transition into this phase is marked by consistent performance of the desired behavior and with high self-efficacy. Unique to this approach is that maintenance is conceived as a penultimate stage in the behavioral pattern. The final stage suggests that repeated behavior becomes a habit (stimulus-driven behavioral response under low awareness), as one achieves peak satisfaction with its performance outcomes.

Humanistic theories of behavior, specifically self-determination theory (Deci and Ryan, 1985, 2000), also have a strong theoretical application in physical activity (Ryan et al., 2009; Teixeira et al., 2012). Like social cognitive approaches, there is no specific mention of behavioral maintenance and corresponding constructs that delineates it from initiation (see Table 1). However, the constructs (i.e., basic need satisfaction) that aid individuals in acquiring the motivation to initiate a behavior are thought to extend to the sustained behavior by contributing to the process of internalization and integration (Deci and Ryan, 2000). Specifically, increased internalization and integration results in a shift from the controlled motivations of behavior initiation (e.g., external regulation) to more autonomous (i.e., intrinsic) motivations expected from sustained behavior.

Application of dual-process models (Deutsch and Strack, 2006), particularly with a focus on affect, have started to increase in physical activity research in recent years (Rhodes et al., 2019). The affective-reflective theory of physical activity (Brand and Ekkekakis, 2018), theory of effort minimization (Cheval et al., 2018), and the affect and health behavior framework (Williams and Evans, 2014) are exemplars of these approaches (see Table 1). No explicit mention of maintenance or maintenance specific constructs are made in these approaches.

Three dual-process approaches that do explicitly include maintenance are the 2 × 2 behavior matrix (Rothman et al., 2009), the maintain IT model (Caldwell et al., 2018), and the physical activity adoption and maintenance model (PAAM; Strobach et al., 2020). In the 2 × 2 behavior matrix, behavioral maintenance is influenced by both automatic and reflective processes. In terms of reflective processes, a key determinant of behavior maintenance is satisfaction with outcomes; specifically, whether prior behavioral experiences are sufficiently satisfying to warrant continued action. In terms of automatic processes, habit is a key determinant in predicting long-term maintenance of a behavior (Rothman et al., 2009). The maintain IT model suggests that behavioral maintenance involves the lowering of executive function resources involved in behavioral enactment over time as a result of the emergence of identities related to the behavior that facilitate swift and less effortful enactment. Finally, the PAAM provides no explicit definition of maintenance, but instead suggests that physical activity patterns over time increasingly rely on implicit processes (e.g., habit, affect) for behavior regulation, with a reciprocal decrease in reliance on explicit processes (e.g., executive functions, trait self-regulations).

Action control models that specifically focus on intention formation and intention translation in physical activity have also seen considerable attention, given the well-recognized gap between intention and action (Rhodes and Yao, 2015). Three common exemplars of action control models in physical activity that discuss maintenance include the health action process approach (HAPA; Schwarzer, 2008), MOVO process model (Fuchs et al., 2012), and multi-process action control (M-PAC; Rhodes, 2017). In the HAPA, post-intentional constructs (i.e., action planning, coping planning, maintenance self-efficacy, recovery self-efficacy) are thought to be stronger amongst individuals who maintain behavior, compared to those who have only initiated a behavior. Similarly, the MOVO process model suggests that goal intention, along with volitional (i.e., post-intentional) constructs like action planning and barrier management are expected to be higher among individuals who can successfully maintain their behavior, compared to those who have only initiated physical activity. In the M-PAC approach, maintenance is described as a result of the development of identity and habit (known as reflexive processes) and their subsequent determination of behavior over time that supplants the need for behavioral self-regulation tactics (known as regulatory processes).

The socioecological approach (Stokols, 1996; Spence and Lee, 2003) to understanding physical activity has also been a dominant theoretical framework in research for over two decades (Rhodes et al., 2019). No explicit mention of maintenance or a maintenance specific construct is made in this approach (see Table 1). Rather, physical activity behavior is thought to be influenced by both psychological factors (e.g., attitudes, self-efficacy) and a cascading series of ecosystems (i.e., microsystem, mesosystem, exosystem, and macrosystem), each of which encompass dimensions from increasingly distal social and physical environments to the individual.

Finally, our literature search for conceptions of maintenance and physical activity identified several approaches that have not seen considerable research, but are worthy of critical analyses on this topic. These included the lapse-recovery relationship of maintenance (Kahlert, 2015), definitional difference between adoption and maintenance of physical activity (Marcus et al., 2000), and the timescale separated model (Spruijt-Metz et al., 2015). According to the lapse-recovery relationship, maintenance is defined by consistent recoveries from behavioral lapses, and a decline in the number of lapses over time. A lapse is defined by not meeting one's personal physical activity goals. Marcus et al. (2000) contend that maintenance is a result of regular physical activity over six-months. By contrast, the timescale separated model proposes that certain constructs associated with behavioral maintenance (e.g., identity, habit) develop by the accrual of repeated short-term physical activity successes.

In summary, the social cognitive, humanistic, and socioecological approaches that have dominated considerable research in physical activity have generally left the definition of maintenance unspecified from initiation. This is not to say that conceptualizations of maintenance are absent. Indeed, many theories denote maintenance as the result of consistent effort and self-regulation (e.g., HAPA, MOVO, lapse-recovery), while others highlight more satisfaction and integration (Four Phases of the Behavior Change Process, SDT). More recent dual-process approaches note more efficiency/automaticity (maintain IT, M-PAC, PAAM) or a hybrid (2x2 Matrix). However, even stage theories, that demark a temporal (i.e., TTM) or reflective (i.e., the four phases model) transition between maintenance and other stages of behavior, adhere to definitions of maintenance that are arbitrary or vague, respectively. Overall, while many theories presuppose a distinction between the processes of initiation and maintenance of behavior through the inclusion of unique determinants or contextual factors that influence maintenance, none provide an explicit definition of what maintenance constitutes.

## Theoretical Constructs Proposed to Determine Maintenance

One of the ways to assist in understanding a behavior is to explore constructs that may determine it (Baranowski et al., 1998). Thus, constructs that are theorized to determine maintenance may be very informative for both a basic science understanding of its processes and an applied focus on how to promote behavioral maintenance. This approach was undertaken in a review by Kwasnicka et al. (2016), coding the constructs of 100 behavioral theories into five general themes that distinctly explain maintenance (see Table 2 for these paraphrased and transcribed themes and constructs). The definition of maintenance used for the review was extremely broad, to include “The continuous performance of a behavior following an initial intentional change at a level that significantly differs from the baseline performance (p. 280).”

**Table 2:** Construct and concepts associated with physical activity maintenance.

**Construct/Concept**	**Definition**	**Relevant theories**	**Contribution to physical activity maintenance example**
**Theme 1: Maintenance motives**			
Enjoyment of behavior and satisfaction with outcomes	The immediate and affective outcomes associated with the performance of a behavior or the positive affective and/or cognitive evaluations associated.	• Regulatory fit theory • Temporal self-regulation theory • Model of behavior maintenance • Groningen active living model	Deriving enjoyment or satisfaction during or immediately after a bout of exercise positively reinforces motivation to maintain exercise in the future.
Self-determination	The intrinsic motivation or inherent satisfaction associated with a behavior, typically as it aligns with an individual's values and personal relevancies.	• Self-determination theory	Feeling accomplished or appropriately challenged by a form or program of physical activity further motivates repeated physical activity in the future.
Identity	A component of an individual's multi-dimensional self-concept or self-representation, informed by social structures and past experiences, that act as personal standards for behavior. Congruence with identity is thought to aid in behavior internalization processes.	• M-PAC approach • Process of reinvention theory • Health behavior internalization model	Self-identifying as an exerciser or as active encourages maintenance of physical activity while also discouraging incongruent behaviors (e.g., excessive sedentary behavior).
**Theme 2: Self-regulation**			
Self-regulation need	The level of self-regulation required to facilitate behavior change. Self-regulation need is thought to reflect motivation, in that moments of low motivation beget higher self-regulation need to facilitate behavior.	• HAPA • Strength model of self-control	Successful maintenance of physical activity requires a higher need for self-regulation if motivation to be physically active is diminished.
Self-regulation skill	An individual's capacity to self-regulate their behavior as their self-regulatory demands vary over time. These skills are often represented through one's planning skills, inhibition control, and task switching.	• Temporal model of self-regulation • Model of self-control	Individuals with higher self-regulatory skill can continue to successfully self-regulate their physical activity behavior as the costs, resources, and barriers to physical activity vary over time.
Self-regulation processes	The ongoing processes of self-monitoring, self-evaluation, self-reinforcement and combatting influences that conflict with long-term goals. Individuals monitor current behavior against their goals and adjust their efforts to ameliorate discrepancies, leading to either satisfaction or dissatisfaction.	• Control theory • Self-regulation theory	Continually identifying and successfully resolving discrepancies between one's current physical activity and a desired physical activity goal facilitates maintenance of physical activity.
Lapse, relapse, and coping with behavioral barriers	A lapse is a singular deviation from the desired goal or behavior. A relapse is a series of lapses, typically resulting in a reversion to prior behavior. Coping with behavioral barriers may involve both confidence in one's ability to respond to behavioral barriers (i.e., coping self-efficacy) and confidence in one's ability to recover from a setback (i.e., recovery self-efficacy).	• HAPA • Relapse prevention theory	Developing coping plans to address potential/actual barriers to regular physical activity can improve coping responses to these barriers, which in turn can improve self-efficacy and outcome expectancies, decreasing the likelihood for future relapse.
**Theme 3: Habits**			
Dual process models and habit theories	Behavior is a function of two systems: a slower and cognitively demanding reflective system; and a quicker, more impulsive automatic system. When psychological resources are low, behavior is defaulted to the automatic system. However, as a behavior is repeated, reliance on the reflective system shifts toward the automatic system, promoting habit formation. Habitual behaviors necessitate minimal reflective system input, requiring few resources or awareness.	• Dual-system models • Health-related model of behavior change • 2 × 2 behavior change matrix • Theory of interpersonal behavior • Habit theory	Repeated successful performances of physical activity behavior(s) can help form physical activity habit(s), through shifting self-regulatory reliance from the reflective system to the automatic system. These physical activity habits require relatively few cognitive resources or awareness, facilitating their maintenance – particularly during times when resources for self-regulation are low.
Learning theories and habit	Repetition and reinforcement form the basis for habit formation (i.e., learning). Concurrent repetition and reinforcement of an unconditioned stimulus with an external stimulus will lead to behavior change maintenance. Factors that promote habit formation include situating new learning in relevant contexts; providing retrieval cues after learning is complete; and varying new contexts of learning. Similar to learning, disassociating (or unlearning) a learned behavior is a slow process.	• A learning theory perspective on the maintenance of behavior change • Classical conditioning • Operant conditioning	Continued repetition of a physical activity behavior with an external stimuli (e.g., going for a walk at lunchtime) can promote habit formation of that physical activity behavior. Conversely, unlearning a competing habitual behavior, such as sedentary behavior, is difficult and may not be possible.
**Theme 4: Resources**			
Self-regulation as a limited resource	Self-regulation (e.g., coping with stress, resisting temptations) draws upon finite mental resources which can be depleted through continued effort, with each subsequent effort more likely to fail. Insufficient efforts to self-regulate will likely default behavioral responses to the automatic system. Mental resources can be replenished through rest and positive affect.	• Strength model of self-control	The compounded self-regulatory effort needed to deal with daily stressors (e.g., work-related stress, refusing a donut at work) drain the pool of mental resources available to self-regulate physical activity behavior later on in the day, which may lead to maintenance failure (e.g., sedentary behavior).
Inter-individual differences in resources and resources availability	Individual and situational differences among moderators of dual system processing (e.g., unconscious positive expectations toward unhealthy behavior, low working memory capacity, influence of substances) can hinder behavior change maintenance through preferential shifts to automatic system. Resource availability is also contingent on individuals' goal selection, which varies over the lifespan.	• Dual system models • Impulsive versus reflective framework • Model of selection, optimisation, and compensation	An individual with unconscious positive expectations toward a default behavior of sitting on the couch and watching TV would require higher cognitive resources to successfully self-regulate behavior and perform physical activity, as compared to an individual without these biases.
**Theme 5: Environment and social influences**			
Environment	Environmental factors determine how much active self-regulation and resources are required for behavior change. Effortful behavior change is less likely to occur in unconducive environments. On the other hand, habitual behaviors developed within a specific environmental context are easier to maintain in the same environment. Changes to the environment present as both threat and opportunity for habitual behavior change and new behavior change, respectively.	• Process model for supporting lifestyle change • 2 × 2 behavior change matrix • Habit theory • Temptation bundling	Attempting to continue a gym-based exercise program at home requires more self-regulation and resources and is likely to not be maintained if the home environment is not structured similarly. Conversely, watching TV solely on the treadmill at the gym presents as an opportunity to form a new habit of physical activity behavior.
Social influence	The effect others have on an individual's opinions, emotional states and behaviors, which can affect the effort needed to perform new behaviors or the capacity to maintain behaviors through aligning one's actions to social norms or standards. The effect of social influence is higher when elicited by trusted persons and people with whom the individual feels connection with.	• Self-determination theory • Social cognitive theory • Social identity model • Substance abuse theory	The support of respected exercise role models, such as personal fitness trainers and exercise partners, can motivate individuals to maintain physical activity.
Social change—how norms are shaped, accepted and maintained	Large-scale behavior change typically achieved through changing the standards of what is acceptable within a specific social group or community. Social change occurs in three steps: implementation, embedding, and sustaining. Through these steps, newly introduced behaviors become social norms and become ‘integrated’ within their social context. Social change is maintained when individuals feel responsible and capable of managing the programmes promoting change.	• Social change theory • Normalization process theory • Theory of change	Altering social norms through social change can improve maintenance of a physical activity behavior through establishing a community with a strong social norm of exercise (e.g., a mom-led after-school running group for moms)

*Table adapted from Kwasnicka et al. (2016)*.

Theme one included the motives that might be associated with maintenance (Kwasnicka et al., 2016). These included broad enjoyment and satisfaction with behavioral outcomes, self-determination, and identity. Theme two, by contrast, focused on the needs, skills, processes, and behaviors that an individual may possess to self-regulate a behavioral action over time. Theme three was specific to habit development, the process of how repeated action within the same context can develop into an automatic behavioral response from preceding environmental cues over time. Theme four represented a collection of constructs that represent psychological assets that can be drawn on to repeatedly perform a behavior under challenging conditions, such as self-control, ego strength/depletion, and goal optimization and selection. The final theme included a collection of constructs that define the social and environmental determinants of continuing a behavior. These constructs, such as the built environment and environmental alternatives, social support, social identity, and social norms were considered important to provide options and opportunity for repeated behavioral responses.

Taken together, the Kwasnicka et al. (2016) review provides a helpful palette of themes that may need to be considered for maintenance, as well as associated constructs within these themes. The review outcomes were still hindered, however, by a lack of definition of maintenance that explicitly distinguishes constructs specific to maintenance from constructs that inform behavioral performance during any point in the behavior change process. In fact, many theories that do not demarcate maintenance from initiation (see Table 1) have considerable construct content coverage among this list (e.g., social and built environment, self-regulation and self-control). The need remains to provide a working definition of maintenance that can assist in delineating core constructs specific to physical activity maintenance and to produce a specific agenda for research moving forward.

## A Working Definition of Physical Activity Maintenance

Based on our critical analysis of key physical activity theories and constructs noted in the prior two sections, we suggest a working definition. We suggest physical activity maintenance be operationalized as a process marked by a shift in the mechanisms of action determining behavioral performance, when contrasted with the mechanisms of action that were required when the behavioral performance was first initiated.

Specifically, we suggest that the process of physical activity maintenance involves a dynamic development of mechanisms of action that engender greater perceived behavioral enactment efficiency that partially supplant prior mechanisms of action that required greater perceived cognitive recourses to enact physical activity.

**Figure 1:**
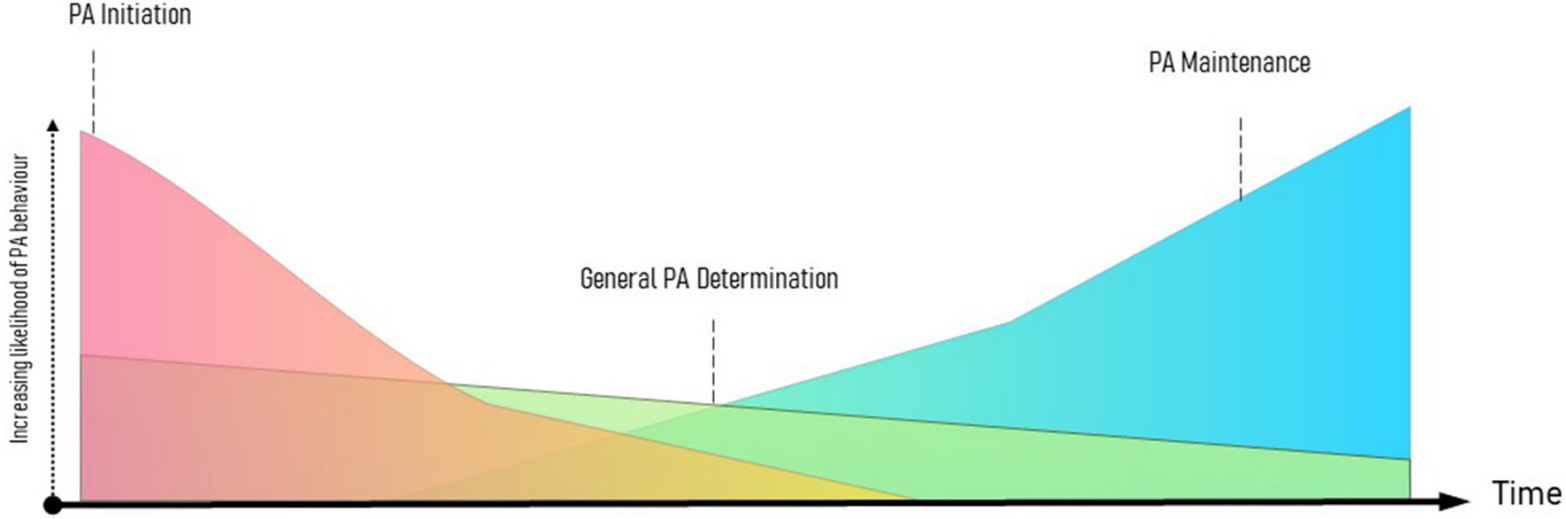
Proposed pattern of physical activity initiation, general determination, and maintenance.

This conceptualization of maintenance is not considered an absolute and characterizable state of behavioral performance, such as a stage. Some constructs that were critical to behavioral performance during initiation will still be important to determining physical activity at later enactments (i.e., general behavioral determinants; see Figure 1). Instead, we propose that the maintenance process adds a layer of determinants to behavioral performance that improve the perceived efficiency of enactment, in combination with constructs already determining physical activity.

Our operationalization of the maintenance process is based on several streams of prior theorizing. First, this operationalization is mindful of Rothman (2000), who suggested maintenance is determined by a shift in expected feelings or outcomes over any mere behavioral performance time marker. Later work by Rothman et al. (2009) further detailed this notion, suggesting that transitions from initiation to maintenance are marked by shifts in an individual's decisional focus from outcome expectancies to satisfaction with outcomes. A later expansion of this theory (Rothman et al., 2011) posited maintenance as a phase of behavior change marked by both successful sustained behavior and the confidence to continue to perform said behavior, in which the individual desires to sustain their new, successful pattern of behavior. Further, this phase of maintenance is distinct from habit—the phase in which behavior is self-perpetuated and no longer actively valuated. However, in contrast to Rothman (2000), Rothman et al. (2011), we do not view maintenance as a phase, with habit formation as an end state.

Our definition of maintenance is also informed by the M-PAC approach (Rhodes, 2017), the maintain IT model (Caldwell et al., 2018), and general assumptions of dual-process models (Deutsch and Strack, 2006; Evans and Frankish, 2009; Kahneman, 2011; Strobach et al., 2020) where more automatic or reflexive behavioral determinants produce behavioral performance efficiency. However, our definition does not propose that only automatic determinants are necessary to produce the maintenance process, as developments in some constructs that are more reflective in nature during repeated behavior may also produce maintenance to the extent that they engender a greater perception of efficiency of action. Further, our proposal that the maintenance process represents a layer of constructs that augment a larger network of behavioral determinants across time, is also reflective of recent research in habit, which acknowledges that complex behaviors like physical activity are not determined by all or nothing processes (Gardner et al., 2016; Rhodes and Rebar, 2018). Instead, complex behaviors like physical activity have preparatory, instigation and execution sub-components (Hagger and Luszczynska, 2014; Phillips and Gardner, 2016; Kaushal et al., 2017) which levy self-regulatory processes and skills, even during long-term behavioral performance. Thus, our definition of maintenance is based on a model where long-standing continuation of physical activity performance can be independent of the maintenance process, although this is unlikely due to the eventual challenge of exhausting cognitive resources (Baumeister et al., 1994; Strack and Deutsch, 2004; Caldwell et al., 2018).

This operationalization of maintenance also allows for more focused theorizing and subsequent falsifiability hypothesis testing of constructs that may determine maintenance. Specifically, determinants of the maintenance process would need to show (1) a change in their absolute value across behavioral performance, and (2) an increase in their magnitude of effect on physical activity over time. Relatedly, determinants of physical activity, independent of an initiation or maintenance process, would show invariance of change in their comparative effects on physical activity over time, and determinants of initiation would show a proportional decrease in their effects on behavior over time. This allows for a fulsome examination of different determinants of physical activity, where time frames of analysis would also be exploratory and likely ideographic to the behavior, and constructs within the theory.

A full understanding of which constructs fulfill the criteria for this definition of maintenance is beyond the scope of this paper, but some of the constructs noted in Kwasnicka et al. (2016) would likely fit this operationalization of maintenance more than others. For example, from the motives theme, satisfaction with the behavior and a shift in self-determined motives (e.g., from external regulation to identified/integrated regulation or intrinsic motivation) would seem possible to account for the maintenance process (Teixeira et al., 2012). Both satisfaction and self-determined forms of motivation require behavioral experience (Ryan et al., 2009; Baldwin and Sala, 2018), and thus it is conceivable (a) satisfaction shifts across time and (b) higher satisfaction and/or self-determined motivation could supplant the cognitive resources needed to engender continued physical activity that is considered unsatisfying and externally regulated (Hagger et al., 2010; Milyavskaya et al., 2015; Huffman et al., 2020).

A similar logic can be used for habit and self- or social-identity as critical constructs that may explain physical activity maintenance. All of these constructs presuppose behavioral performance experiences as a pre-requisite (Rhodes, 2017), which supports the theory that these variables are dynamic and develop over time from behavioral initiation (Spruijt-Metz et al., 2015). Habit theory suggests that the learned cue-behavior conditioning that forms habits reduces the requirement for effortful self-regulation (Wood and Runger, 2016) so this directly supports our definition of a determinant of the maintenance process. Similarly, self- and social-identity are considered reflexive regulating systems, that reduce the burden of effortful self-regulation and executive function (Hogg and Abrams, 1988; Stryker and Burke, 2000), so these also align with the maintenance process. Specifically, as individuals' experiences with physical activity begin to shift beliefs as a result of successful behavioral enactment (Rothman et al., 2009, 2011), so too does their identity change to support its maintenance (Epiphaniou and Ogden, 2010). Drawing upon dual-process theories, the development of both habit and identity as reflexive (i.e., Type I) processes, over time, should reduce the burden upon effortful self-regulation and executive function (i.e., Type II) processes related to physical activity maintenance (Rhodes, 2017; Caldwell et al., 2018; Strobach et al., 2020).

By contrast, we suggest that constructs related to the themes of self-regulation (i.e., Theme 2) and resources (i.e., Theme 4) in Kwasnicka et al. (2016) are likely not determinants of the maintenance process, but instead these are important general determinants of physical activity. Specifically, self-regulation and self-control are mindful and effortful processes that would not evoke perceived efficiency in continued behavioral enactment, despite their importance to behavioral engagement (Nigg et al., 2008; Kahlert, 2015). In support of this are the TTM's “overt” behavioral processes, like social support and stimulus control, which describe processes that must be implemented successfully throughout the stages of change (Paxton et al., 2008). Similar evidence from studies utilizing the HAPA demonstrate similar path coefficients for action and coping planning among both intenders and actors (Lippke et al., 2004). Further, a review by van Stralen et al. (2009) found that, among older adults, self-regulatory strategies of action planning and coping planning were positively associated with both physical activity initiation and maintenance. We also suggest that static constructs, such as many constructs of the social and built environment that are not dynamically changing across physical activity experiences, are likely not linked to our definition of behavioral maintenance—at least not as direct mechanisms. These constructs do not shift over time; instead, they would likely determine general behavioral continuance. It is entirely possible, however, that the built and social environment facilitate or inhibit (e.g., promote or inhibit habit formation, promote or inhibit satisfaction and self-determination shifts, increase or decrease the need for self-regulation and self control) other constructs germane to the maintenance process.

## Future Directions

**Figure 2:**
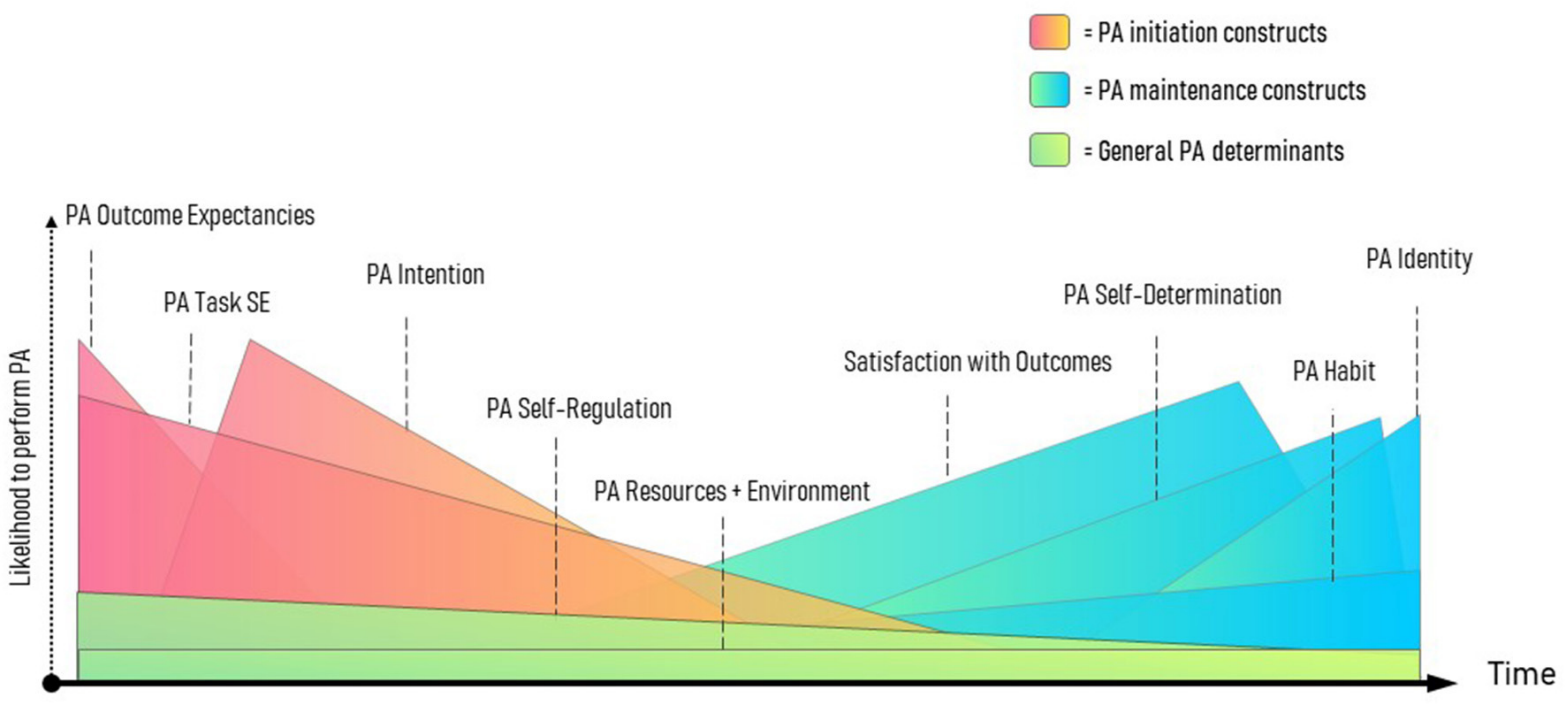
Proposed pattern of physical activity initiation, general determination, and maintenance and associated constructs. Constructs are mere prototype examples and not established in this order.

The operationalization of maintenance provided above allows for several future directions, all of which can be tested for either support or falsification. Importantly, this definition of maintenance allows for more precise testing of whether a construct represents a maintenance mechanism of action. However, it is important to note that while this conceptual paper provides a testable operationalization of maintenance, the validity of this conception is entirely dependent on future evidence. In the section above, we suggested some constructs from Kwasnicka et al. (2016) that may, on first appraisal, be the most likely determinants of the maintenance process, yet this can be specifically tested through longitudinal exploration of change and predictive capability of physical activity over time. More recent advances in intensive longitudinal analyses methods (Bolger and Laurenceau, 2013) can determine the necessary conditions that a construct has changed in absolute value as a consequence of ongoing behavioral performance and had greater predictive value upon ongoing behavior (Carpenter et al., 2016; Dunton, 2018). In fact, commensurate with Figure 2, one could specifically plot constructs that represent determinants of physical activity generally, from those that determine the maintenance process.

One of the most important questions about constructs that have been theoretically associated with maintenance, such as habit (Verplanken and Aarts, 1999), is the period of time involved in reaching this peak (Lally et al., 2009; Kaushal and Rhodes, 2015; Keller et al., 2021). This allows for the development of theory in physical activity behavior maintenance, but it also has tremendous practical value for promoters. Specifically, those who promote physical activity can adjust behavior change techniques to foster maintenance, with a firmer understanding of the length of time that clients may need to invest in the intensive cognitive resources during early behavior change initiation and continuation. A clear definition of maintenance allows researchers to expedite tests of peak maintenance responses. Furthermore, our operationalization of maintenance allows for an exploration of how different determinants (e.g., habit vs. identity) of maintenance may differ in the time to reach peak, as well as whether promoting certain combinations (e.g., habit and satisfaction) of maintenance determinants expedite the process of peak maintenance.

As noted above, this operationalization of maintenance should be helpful in delineating critical mechanisms of action; however, future research focusing on key moderators of the maintenance process is also warranted. Specifically, an understanding for whom, under what conditions, is the maintenance process most likely to occur is likely essential information for physical activity practitioners. Relatedly, it seems entirely likely that non-physical activity habits (Gardner et al., 2021), priorities (Conner et al., 2016), and goals (Rhodes et al., 2016) as well as the social and physical environments that foster them could differentiate the facilitating conditions for those who are able to develop physical activity maintenance compared to those who have more difficulty. Use of the operating conditions framework (Rothman and Sheeran, 2020) is recommended as a tool to consider these contextual moderators that are likely critical in forming the maintenance process.

A definition of maintenance, independent of a behavioral timeline, may also help pave research to create a more specific definition of the process of initiation. While the focus of this paper has been on maintenance, the approach toward our definition could also be applied to understand initiation, whereby there is a shift in the magnitude of specific determinants that lead to beginning physical activity that decline after its initial performance (see Figure 1). Taken together, this may assist in a comprehensive approach to physical activity promotion, which leads toward key constructs and behavior change techniques necessary to the initiation process, general behavioral continuation, and the maintenance process of behavior.

Finally, because the definition of maintenance in this paper delineates a psychological process, under a pre-requisite of a continued pattern of physical activity, this supports the possibility of creating a perceptual measure of maintenance that is sensitive to the fluctuations in perceived efficiency of physical activity across time. A focus on the volitional self-regulation (Marlatt and George, 1984; Schwarzer, 2008), self-control (Baumeister, 2003), and dual process (Strack and Deutsch, 2004) literature would be helpful to adapt a basic assessment of perceptual shift in the cognitive recourses required to engage in continued physical activity that demarcates the definition of maintenance put forward in this paper. Scale creation and validity assessment procedures (Messick, 1995) for a measure of maintenance across longitudinal sampling would be the logical future direction for this endeavor.

In conclusion, the purpose of this critical review was to propose a working definition of physical activity maintenance, followed by recommendations for future research. While a clear basic and applied rationale has been made for the differences between physical activity initiation and maintenance, a definition independent of behavioral performance across an arbitrary time-frame, has likely impaired the advancement of theory and practice. To develop our operationalization of maintenance, we first overviewed the conception of maintenance and co-requisite theoretical constructs in theories used in physical activity research. Based on this information, we suggest physical activity maintenance is a process marked by a shift in the mechanisms of action determining behavioral performance, which engender greater perceived behavioral enactment efficiency. Based on this definition, we then proposed a method of falsifiability hypothesis testing of theoretical constructs that may determine the maintenance process from those constructs that may be critical to physical activity participation more generally. Finally, our review concluded with future research suggestions such as building a measure of maintenance, examining key constructs that may determine maintenance, conditions and time frames associated with maintenance development, and testing for how initiation, general physical activity determination, and maintenance may interrelate in physical activity behavior change.

## Author Contributions

RR was the primary writer of the paper. WS extracted information for the tables, created the figures, and edited the main text. Both authors consulted on all aspects of the conceptual paper.

## Conflict of Interest

The authors declare that the research was conducted in the absence of any commercial or financial relationships that could be construed as a potential conflict of interest. The handling editor declared a past co-authorship with one of the authors RR.

## Publisher's Note

All claims expressed in this article are solely those of the authors and do not necessarily represent those of their affiliated organizations, or those of the publisher, the editors and the reviewers. Any product that may be evaluated in this article, or claim that may be made by its manufacturer, is not guaranteed or endorsed by the publisher.
