# Supplementary material for: Physical Activity Maintenance: A Critical Narrative Review and Directions for Future Research
Source: Front Psychol. 2021 Sep 6;12:725671. doi: 10.3389/fpsyg.2021.725671 (PMC8450373; doi:10.3389/fpsyg.2021.725671)
Supplement: Supplementary file 1 [file Table_1.DOCX]

**Identification of studies via databases**

Records identified from:

Databases: Google Scholar (n = 50), Pubmed (n=50), Scopus (n = 50)

Systematic reviews (n = 5)

Rhodes et al. (2019)

**Identification**

Records screened

(n = 182)

Reports sought for retrieval

(n = 29)

**Screening**

Reports assessed for theories and conceptualizations

(n = 29)

Theories/conceptualizations included in critical narrative review

(n = 20)

**Included**
