# Supplementary material for: Physical Activity Maintenance: A Critical Narrative Review and Directions for Future Research
Source: Front Psychol. 2021 Sep 6;12:725671. doi: 10.3389/fpsyg.2021.725671 (PMC8450373; doi:10.3389/fpsyg.2021.725671)
Supplement: Supplementary file 2 [file Table_2.DOCX]

**Supplementary Material 1.**

*Scale for the Assessment of Narrative Review Articles Checklist*

| Aspect of Quality | Reference in paper |
| --- | --- |
| Justification of the article’s importance for the readership | The present work reviews the physical activity maintenance literature and highlights the lack of a clear, measurable definition of physical activity maintenance. Further, the present paper contextualizes previous definitions of physical activity maintenance and puts forth a working definition that can be tested and validated by future work. |
| Statement of concrete aims or formulation of questions | Statement of the specific purpose of the narrative review are described on pages 4 and 5. |
| Description of the literature search | Details regarding the literature search strategy, databases searched, and number of results are described on pages 5 and 6, and by the PRISMA flowchart (Supplemental Figure 1). |
| Referencing | All key arguments and theoretical postulates have been supported by references. |
| Scientific reasoning | High quality evidence and systematic reviews are referenced with respect to theoretical concepts, throughout the study. |
| Appropriate presentation of data | Given the theoretical and conceptual nature of this narrative review, outcomes examined and presented relate to associations between behavioural constructs and physical activity maintenance, as well as moderators and mediators of this behaviour. |
| *Note.* Table adapted from Baethge, C., Goldbeck-Wood, S., & Mertens, S. (2019). SANRA—a scale for the quality assessment of narrative review articles*. Research Integrity and Peer Review, 4*, 5. | |
